# Serum Samples from Co-Infected and Domestic Cat Field Isolates Nonspecifically Bind FIV and Other Antigens in Enzyme-Linked Immunosorbent Assays

**DOI:** 10.3390/pathogens10060665

**Published:** 2021-05-28

**Authors:** Alex Moskaluk, Mary Nehring, Sue VandeWoude

**Affiliations:** Microbiology, Immunology and Pathology, Colorado State University, Fort Collins, CO 80523, USA; alex.moskaluk@colostate.edu (A.M.); mnehring@colostate.edu (M.N.)

**Keywords:** enzyme-linked immunosorbent assays, ELISAs, feline immunodeficiency virus, feline foamy virus, FIV, FFV

## Abstract

We evaluated enzyme-linked immunosorbent assay (ELISA) specificity for measuring seroantibody responses to two types of retroviral infections in domestic cats: feline immunodeficiency virus (FIV) and feline foamy virus (FFV). We compared the seroreactivity of specific pathogen-free (SPF) cat sera, sera from SPF cats inoculated with either FIV or FFV, and field isolates (e.g., shelter or privately owned cats). Sera from SPF cats experimentally infected with the cognate virus had significantly lower background in both FIV and FFV ELISAs compared to sera from negative field isolates. ELISA values for SPF cats exposed to either FIV or FFV tended to have higher OD values on the opposite ELISA antigen plate. FIV nonspecific background absorbance was greater than that of FFV, and 10 of 15 sera samples from FIV seronegative field samples were measured in the indeterminant range. These findings highlight that exposure to off-target pathogens elicit antibodies that may nonspecifically bind to antigens used in binding assays; therefore, validation using sera from SPF animals exposed during controlled infection results in the setting of a cutoff value that may be inappropriately low when applied to field samples. Our work also suggests that infection of domestic cats with pathogens other than FIV results in antibodies that cross-react with the FIV Gag antigen.

## 1. Introduction

Serologic assays are routinely used for evidence of exposure to pathogens, typically by assessing antibodies to a specific antigenic epitope to the pathogen of interest in blood samples collected from individuals or populations of humans or animals [1]. An ideal assay has high sensitivity—the ability to detect low antibody titers for a targeted pathogen with few false negative results—and high specificity—the capacity to distinguish antibodies raised against the pathogen of interest with few false positive results. Assay validation typically requires use of samples from known seropositive and seronegative individuals so that accurate sensitivity and specificity can be calculated.

The phenomenon of nonspecific binding in solid-phase immunoassays has been recognized and is associated with primary or secondary antibody binding to nontarget antigens or surfaces, high immunoglobulin levels, or inflammatory markers [1,2]. Identifying nonspecific binding is crucial for developing novel serologic assays as this type of binding can falsely lead to elevated levels of the analyte measured [3]. This can cause results to fall within the indeterminate range, or, with high enough nonspecific binding, can result in false positive reactions. For viral infections, it has been shown that antibodies to other viruses can interfere with the detection of the virus of interest in solid-phase immunoassays [4]. In order to achieve highest specificity, serologic assays used for assessment of prior infections require the use of appropriate samples to account for potential nonspecific binding.

Feline immunodeficiency virus (FIV) is a T-cell tropic lentivirus with worldwide distribution in cats [5]. FIV infects CD4-T and other white blood cells, and results in CD4/CD8 T cell inversion. Lymphoma, gingivitis, neurologic diseases, and opportunistic infections are common sequelae [5]. The course of the infection is variable, but infected animals remain persistently infected for life. Evidence of infection is typically measured by commercially available or point-of-care serologic assays [6]. Feline foamy virus (FFV) is a spumavirus also resulting in lifelong infection in cats. FFV infection is common in the United States and has not been associated with apparent clinical disease [7,8]. Commercially available assays are not readily available to detect FFV infection in domestic cats, despite its high prevalence.

We developed standard antigen-enzyme-linked immunosorbent assay (ELISA) serologic detection assays for the assessment of exposure to FIV and FFV. Use of serum from an unvaccinated, specific pathogen-free (SPF) cat colony as negative controls and cats experimentally infected with FIV, FFV, or vaccinated with a commercially available FIV vaccine as positive controls repeatedly resulted in 100% sensitivity and specificity of our assay systems following seroconversion four weeks after viral exposure. We took advantage of a unique bioarchive containing well-characterized field and experimental serum samples to compare ELISA binding characteristics of known positive and negative individuals on plates validated with ‘gold standard’ serum samples. Our findings clearly document significantly higher nonspecific binding in samples obtained from animals with at least one other documented viral infection, particularly for FIV antigens.

## 2. Results

In total, 56 individual serum samples were tested (31 samples for FIV and 61 samples for FFV), (Table 1). These included samples from SPF cats exposed to tissue culture supernatant without virus (Sham); SPF cats inadvertently infected with feline parvovirus (SPF FPV+); SPF cats experimentally infected with FIV, FFV, or vaccinated with FIV; and several cats from ‘field’ situations that had been extensively screened for common viral infections.

### 2.1. FIV SPF, Sham and Field Negative Results

**Figure 1:**
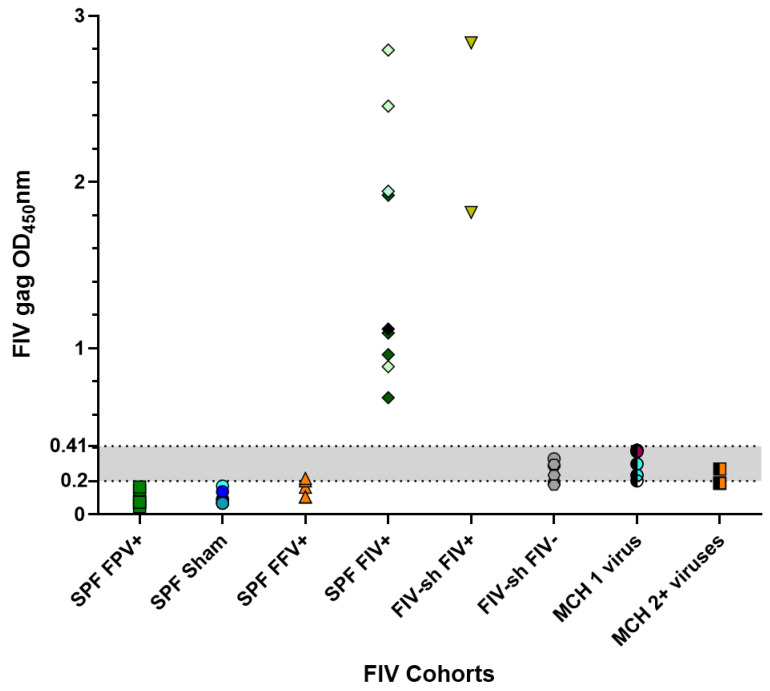
Sera from field cats and SPF cats infected with FFV had higher nonspecific binding in an FIV ELISA assay. Serum samples from SPF cats with (1) FPV infection (SPF FPV+) (n = 6); (2) sham inoculations (SPF Sham) (n = 3; 1 sample run on 2 plates = light blue, 1 sample run on 4 plates = dark blue); (3) FFV infection (SPF FFV+) (n = 5); or (4) FIV infections (SPF FIV+) (n = 3; 1 sample run on 2 plates = black, 1 sample run on 3 plates = dark green, 1 sample run on 4 plates = light green) and serum samples from (5) multi-cat household cats with FIV infections (FIV-sh FIV+) (n = 2); (6) multi-cat household cats without FIV infections (FIV-sh FIV–) (n = 7); (7) private colony cats with 1 viral infection (not FIV) (MCH 1 virus) (n = 3; 2 samples run on 2 plates = red or blue); or (8) private colony cats with 2 or more viral infections (not FIV) (MCH 2+ viruses) (n = 2; 1 sample run on 2 plates = orange) were collected as indicated in the text. FIV antibody reactivity was measured as described in the Methods. The indeterminate range (gray shaded area) was calculated as 1–2× the cutoff value. An OD_450_nm value greater than 0.41 was regarded as positive.

The FIV ELISA indeterminant range optical density (OD) value was set within the range of 0.2–0.41 based upon ELISA value measurements from sera from three SPF cats that were FPV negative (Sham cats). All SPF cats had absorbance values below the indeterminant range (Figure 1). SPF FPV+ cat serum had an average OD value of 0.095 on FIV ELISA plates, while Sham-inoculated cats had an average OD value of 0.099 (*p*-value 0.87, Table 2 and Table 3). FIV negative samples collected from field situations had an average OD value of 0.23, significantly higher than that of SPF FPV+ or Sham sera (*p*-value < 0.001, Table 2 and Table 3). In total, 12 of 20 cats (60%), which included 4 FIV negative cats from a shelter, 5 private colony cats that were FIV negative and had 1 other viral infection, 1 private colony cat that was FIV negative and had 2 other viral infections, and 2 SPF FFV inoculated cats also fell within this indeterminate range (Table 2, Figure 1).

### 2.2. FFV SPF, Sham and Field Negative Results

**Figure 2:**
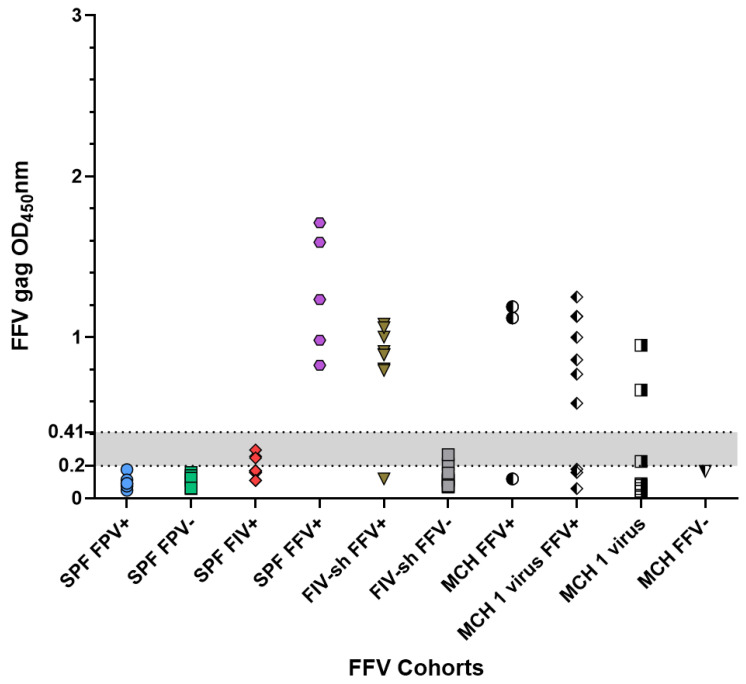
Sera from field cats and SPF cats infected with FIV had higher nonspecific binding in an FFV ELISA assay. Serum samples from SPF cats with (1) FPV infection (SPF FPV+) (n = 6); (2) FFV Sham and FIV and FFV prebleeds (SPF FPV−) (n = 8); (3) FIV infection (SPF FIV+) (n = 3 from 4 timepoints); and (4) FFV infections (SPF FFV+) (n = 5), and serum samples from (5) multi-cat household cats with FFV infections (FIV-sh FFV+) (n = 9); (6) multi-cat household cats without FFV infections (FIV-sh FFV−) (n = 9); (7) private colony cats with FFV infections (MCH FFV+) (n = 3); (8) private colony cats with FFV infections plus 1 viral infection (MCH 1 virus FFV+) (n = 10); (9) private colony cats with 1 viral infection (not FFV) (MCH 1 virus) (n = 10); or (10) private colony cats without FFV infections (MCH FFV−) (n = 1) were collected. FFV antibody reactivity was measured as described in the Methods. The indeterminate range (gray shaded area) was calculated as 1–2× the cutoff value. An OD_450_nm value greater than 0.42 was regarded as positive. In total, 3 out of 12 samples infected with FIV had higher background than SPF FPV+ or Sham cats falling within the indeterminate range, increasing cross-reactivity.

The FFV ELISA indeterminate range of 0.21–0.42 was calculated using values generated from sera from eight FPV negative SPF cats (FFV Sham (n = 3), FIV prebleed (n = 3), and FFV prebleed (n = 2)). All SPF cats had absorbance values below the indeterminant range (Figure 2). Field samples that were FFV negative had an average OD value of 0.19 (Table 2). SPF FPV+ cat sera tested by FFV ELISA had average OD values of 0.09, while Sham cats (n = 3 FFV sham cats) ELISA values averaged OD value of 0.08 (*p*-value 0.66, Table 2 and Table 3). OD values for SPF cats trended lower than field sampled negative cats (*p* = 0.098). The seroreactivity of field negative samples was lower on the FFV ELISA than that noted with FIV ELISA. Overall, 2 FFV negative shelter cats, 3 SPF FIV inoculated cats, and 1 private colony cat that was FFV negative had absorbance values within the indeterminate range (Table 2, Figure 2).

### 2.3. Experimentally Inoculated or Vaccinated FIV and FFV Results

**Figure 3:**
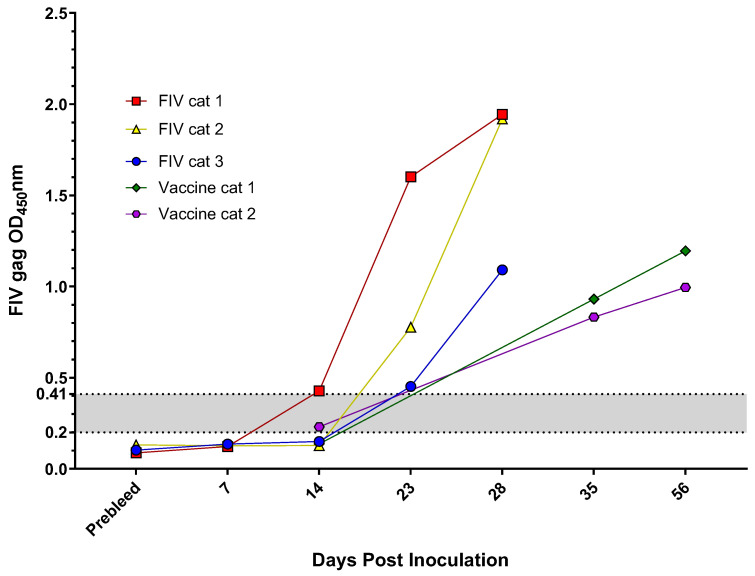
FIV anti-Gag antibody reactivity increased post-infection and post-vaccination. SPF cats were experimentally inoculated with FIV (n = 3) or FIV vaccine (n = 2), and sequential serum samples were collected. FIV antibody reactivity was measured as described in the Methods. The indeterminate range (gray shaded area) was calculated as 1–2× the cutoff value. An OD_450_nm value greater than 0.41 was regarded as positive.

**Figure 4:**
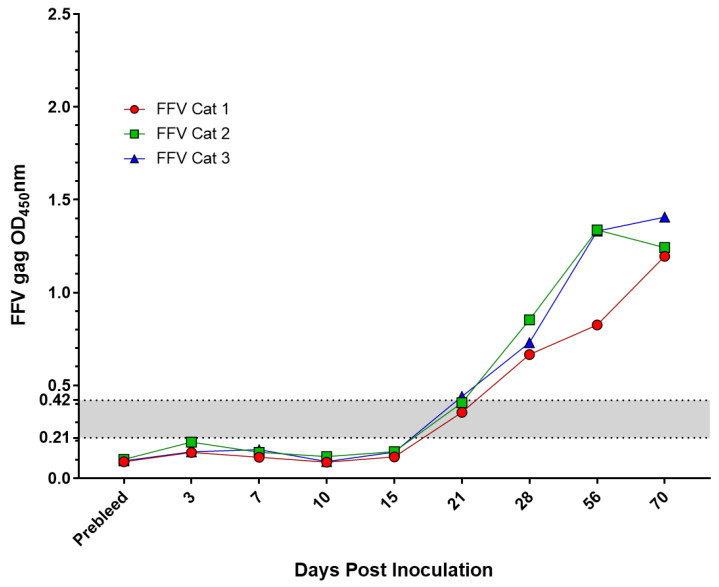
FFV antibodies increased post-infection. SPF cats (n = 3) were inoculated with feline foamy virus (FFV) and sequential serum samples were collected. FFV antibody reactivity was measured as described in the Methods. The indeterminate range (gray shaded area) was calculated as 1–2× the cutoff value. An OD_450_nm value greater than 0.42 was regarded as positive.

SPF cats that were experimentally inoculated with either FIV or FFV demonstrated an increased antibody response over time, as anticipated (Figure 3 and Figure 4). Cats inoculated with FIV had ELISA results indistinguishable from those of seronegative cats at 7 days post infection (DPI). ELISA values for cats at 14 and 23 DPI typically had ELISA values that were close to the range that corresponded with indeterminate samples. By 28 DPI, however, all three FIV cats had a strong antibody response. FFV infected cats had a similar seroconversion time course, though cats tended to seroconvert by 21 DPI. All time points tested after 28 DPI exhibited high levels of antibodies characteristic of infection (Figure 4). SPF cats that were vaccinated for FIV had an increase in antibodies over time. At 14 DPI, all vaccinates demonstrated OD values indicative of FIV infection and comparable to seroconverted experimentally infected cats (Figure 3).

### 2.4. Various Blocking Solutions Did Not Have an Effect on Nonspecific Binding in FIV ELISA

Attempts at enhancing specificity for field samples without reducing sensitivity by using 5% nonfat milk, 1% casein, and 1:200 anti-*E. coli* antibody in the blocking steps of the ELISA did not result in decreases in nonspecific binding as compared to using 2% bovine serum albumin (BSA).

## 3. Discussion

An FIV diagnosis is dependent upon accurate diagnostic testing. As FIV causes antibody titers to be persistently high and FIV polymerase chain reaction (PCR) is unreliable due to variation in the FIV genetic sequence, serology is the preferred method for diagnosis [13]. Antibodies are generated to FIV env, Gag, and pol epitopes of FIV’s env and Gag proteins [14]. Despite high antibody titers, cats remain FIV-infected for life [15]. Anti-Gag IgG levels have been shown to be correlated to the FIV viral load [16]. Prior studies have indicated that 1.8–21.8% IgG antibodies generated during FIV infection specifically bind to capsid and surface FIV proteins [16]. The potential for nonspecific IgG generated during infection to cross-react with other pathogens is currently unknown [16].

Feline foamy virus (FFV) is a retrovirus belonging to the family Spumaretrovirus [17]. The virus is believed to be apathogenic [8,18] and is thought to spread through direct contact via social contacts such as grooming, in contrast to the aggressive interactions that are required to transmit FIV [19]. Once infected, cats have a lifelong infection [20]. FFV antibody detection through ELISA has proven to be a valid method for diagnostic screening [21,22].

This study aimed to evaluate the sensitivity and specificity of in-house laboratory diagnostic ELISAs for FIV and FFV comparing sera from SPF cats with a variety of known viral infections and field samples that had been well characterized. Sera from experimentally infected cats and SPF cats or sham inoculated controls were used to set positive cutoff points and indicated 100% sensitivity and specificity after the 1-month post-inoculation seroconversion period. Cats in the process of seroconverting (i.e., <4 weeks post experimental exposure) had ELISA OD absorbance values that fell within or below the ‘indeterminant range’ defined for each assay.

Following inadvertent infection of SPF cats with feline parvovirus or when testing field isolates from domestic cats from a variety of housing settings, we observed ELISA OD values in the indeterminant range in 22.4% of cases (13/58). We also tested SPF cats infected with FIV or FFV on the converse ELISA protocol to assess the potential for one retroviral infection to induce antibodies that cross-react with antigens from a different retrovirus. Use of SPF cat sera as a negative control and sera from cats experimentally inoculated with laboratory strains of FIV or FFV (i.e., ‘gold standard’ samples) provided an idealized parameterization of the diagnostic assays. We found that ELISA absorbance values generated from serum from cats with irrelevant viral exposures often exceeded cutoff values generated with ‘gold standard’ samples. This resulted in high numbers of indeterminant or false positive findings when analyzing field samples.

The most obvious explanation for the variation seen in the background between field and laboratory cats is the SPF cats lack of exposure to field pathogens and a single exposure to a large amount of virus, resulting in a very specific antibody response with low background. Cat serum samples collected from multi-cat households or animals surrendered to shelters have the potential to be infected with multiple agents and have a much broader repertoire of polyclonal antibodies against a variety of agents, resulting in a significantly higher background. SPF cats with FFV or FPV tended to have lower cross-reactivity to FIV than samples from the private breeding colony and multi-cat household. Thus, cats with multiple pathogen exposures and ongoing infections were more likely to nonspecifically bind FIV Gag antigens than SPF cats with one viral infection. In contrast, 3 out of 12 SPF cats with FIV infection had higher seroreactivity to FFV Gag antigen and only 7.32% of FFV field samples had nonspecific binding. The higher propensity for reactivity to FIV Gag antigen than FFV Gag may be a reflection of antibody affinity or antigen preparation quality or may be a true reflection of the comparative likelihood for development of cross-reactive antibodies to these agents. The bioarchive used in this study was limited in the total number of samples as well as number of samples between groups. Testing more samples from cats with known experimental or field exposures against a battery of antigens would help to reveal more about specific mechanisms underlying cross reactive antibody production.

Analysis of early timepoints in experimentally exposed animals demonstrated that early infections are a second explanation for indeterminant test results, reinforcing that paired serum samples collected 2–4 weeks apart should be able to distinguish indeterminant, nonspecific reactivity from early seroconversion. FIV vaccination produces an antibody signal over time similar to that of cats experimentally infected with FIV, leading to a false-positive FIV infection diagnosis if vaccine history is not considered [6]. We demonstrate here that FIV vaccination can result in absorbance results within the indeterminate range. Retesting cats that fall within the indeterminate range at 2 weeks can help elucidate the cat’s viral status to rule out other viral infections and vaccination/infection [6,23].

In attempts to reduce nonspecific binding on the FIV ELISA, we tested different blocking reagents including 2% BSA, 5% nonfat milk, 1% casein, and 1:200 anti-*E. coli* antibody using the same ELISA protocol for each reagent. While only a few samples were tested, we found no difference between the blocking reagents in reducing nonspecific binding. Given that the samples tested were from cats that had either a current or previous viral infection (other than FIV), we suspect these cats have antibodies that can interact with the FIV Gag antigen.

While the outcomes of this study are not surprising, use of serum from SPF and experimentally animals in comparison to field isolates provided a unique opportunity to conclusively illustrate basic assumptions about sera cross-reactivity that are not readily tested in humans or other species where control of disease exposure is feasible. Our results demonstrate that validation of FIV and other serologic assays using serum from SPF animals using standard cutoff value calculations may lead to false positives. Conversely, the use of field negative serum could result in setting thresholds that overestimate background reactivity, leading to loss of assay sensitivity. Here we used the method of three standard deviations that is a common approach to cut-point setting [24]. In cases where the standard derivation cannot be estimated due to low number of negative samples, other methods of cut-point determination are recommended. For example, a method that uses the ratio of the average sample OD (P) over the average negative sample OD (N) can be used to develop a global cutoff point [25].

This report illustrates that diagnostic assay validation using samples from individuals without exposure to antigens or pathogens may result in overestimation of sensitivity and specificity, particularly during FIV infection. Further, FIV vaccination results may result in false positive or suspect results. Setting an indeterminate range and subjecting suspect samples to additional screening tests is a potential strategy for optimizing sensitivity and specificity. Retesting paired samples 3 weeks following testing offers an option for accurate diagnosis.

## 4. Materials and Methods

### 4.1. Samples

Negative serum samples were collected from 6 unvaccinated, specific pathogen-free (SPF) cats maintained in a breeding colony with restricted housing environment at Colorado State University. Cats were kept in an Association for Assessment and Accreditation of Laboratory Animal Care International-accredited animal facility and were routinely assessed for exposure to feline leukemia virus (FeLV), feline immunodeficiency virus (FIV), feline parvovirus (FPV), calicivirus, feline herpesvirus (Rhinotr), *Giardia*, *Cryptosporidium*, feline coronavirus (FeCOV), felis catus gammaherpesvirus 1 (FcaGHV1), and feline foamy virus (FFV). Serologic testing for FPV was conducted in 21% of the colony in 2014 and 2018 by a commercial laboratory (Cornell University, Animal Health Diagnostic Center, Ithaca, NY, USA) and using in-house qPCR (data not shown). Blood was collected via the jugular or cephalic vein without sedation. All sera collected in 2014 were negative, whereas all samples were strongly positive in 2018. SPF FPV positive cats unassigned to experimental studies (n = 6), FFV prebleeds (n = 2), FIV prebleeds (n = 3), and SPF cats exposed to sterile tissue culture fluid (MYA-1 cells or CrFK culture media) during experimental studies (‘Sham’ cats, n = 3 from FIV studies, n = 3 from FFV studies) were used as negative controls for both FIV and FFV ELISA tests [9,10].

Serum samples were collected from 3 cats infected with FIV and FFV in experimental studies at days 7, 14, 23, and 28 post-infection and days 3, 7, 10, 15, 21, 28, 56, and 70 post-infection, respectively, as previously described [8,9,10]. Negative controls included SPF cats prior to FIV and FFV inoculation and sham inoculates. (Table 1).

Field serum samples were obtained from 2 sources. FIV shelter positive included cats (n = 44 total, n = 9 cats tested by FIV ELISA, n = 18 cats tested by FFV ELISA) from 2 shelter settings characterized as FIV positive by FIV virus isolation and serology [11]. The private multi-cat household (MCH) included cats from a multi-cat FIV household (n = 65 cats total; n = 5 cats tested by FIV ELISA, n = 23 cats tested by FFV ELISA) determined as FIV antibody negative by a commercial ELISA test for FIV, but confirmed positive for either FeLV, FPV, FeCOV, and FFV by a variety of assay methods previously described [11].

Two CSU SPF cats were vaccinated with a commercially available FIV vaccine, Fel-O-Vax (Boehringer Ingelheim, North Ryde, NSW, Australia). Serum samples were collected at 14, 35 and 56 days post vaccination.

### 4.2. FIV and FFV Antigen Preparation

The FIV Gag capsid antigen was prepared as previously described [16]. The FFV Gag capsid antigen (Genbank accession number NC_039242, gene 1442-2986) was prepared by a commercial laboratory (GenScript, Piscataway, NJ, USA) using an amino acid sequence derived from previously published work [21].

### 4.3. ELISA Protocol

High binding plates (Clear Flat-Bottom Immuno Nonsterile 96-Well Plates, Immulon 2 HB, Fisher Scientific, Houston, TX, USA) were coated with either FIV Gag or FFV Gag (100 ng/well) and incubated overnight at 4 °C. Plates were decanted, and 290 µL of 2% BSA in wash buffer were added to each well for 2.5 h at room temperature. Wash buffer (2 mM imidazole, 160 mM NaCl, 0.5 mM EDTA, pH 7.4) was used to remove unbound proteins from the wells. Feline serum was diluted 1:100 in ELISA diluent and added to the wells and incubated for 2 h at room temperature. Samples were run in triplicate with the average between wells determining the OD value. Wells were washed 5 times with wash buffer containing 0.2% Tween. A total of 100 µL of diluted goat anti-cat IgG peroxidase conjugate (diluted 1:5000 in ELISA diluent with 5% mouse sera) was added to each well and incubated for 1 h at room temperature and washed 5 times with wash buffer containing 0.2% Tween. Then, 100 µL of TMB were added to the wells. The reaction was stopped at 10 min with 50 µL of 2.5 N H_2_SO_4_ and read at 450 nm using a Multiskan^®^ Spectrum spectrophotometer (Thermo Fisher).

### 4.4. Calculation of OD Indicating Seropositivity

The cutoff value indicating a positive reaction was determined using the equation

Cutoff = (Average OD_NEG_) + (3×STD OD_NEG_)

where OD_NEG_ represents the OD values of the SPF cats and STD OD_NEG_ indicates the standard deviation between the SPF cats’ OD values [26].

### 4.5. Statistical Analysis

Samples with OD readings between 1 and 2× the cutoff value were considered to be in the indeterminate range. OD values between the sample groups were compared using independent *t*-test (IBM SPSS^©^) assuming equal variance via Levene’s test, where *p*-values above 0.05 were considered not significant.

### 4.6. Blocking Experiments

In order to reduce nonspecific binding, different blocking reagents were tested for the FIV ELISA. The reagents that were utilized were 2% BSA, 5% nonfat milk, 1% casein, and 1:200 anti-*E. coli* antibody. The FIV ELISAs were run as described in the ELISA protocol, only modifying which blocking reagent was utilized. The BSA, nonfat milk, and casein were tested using 3 SPF FPV+ cat serum samples. The anti-*E. coli* antibody was tested using 4 field cat serum samples.

## Figures and Tables

**Table 1:** Feline samples used in this study were acquired from cats with a wide variety of well-characterized virus exposures.

Sample Type	Sample Archive	Description	FIV ELISA	FFV ELISA	Reference (s)
Laboratory Cats	SPF FPV+	Cats from a closed specific pathogen-free breeding colony naturally and inadvertently exposed to feline parvovirus.	6	6	[8,9,10]
	SPF Sham	SPF cats in an FIV naïve control arm receiving culture supernatant from un-infected MYA-1 cells 34 days prior to sampling. SPF cats in the FFV naïve control arm received FFV-negative CrFK culture media 10 days prior to sampling.	3	3	[9,10]
	SPF FPV-	SPF cats prior to FPV exposure	0	5	[9,10]
	SPF FIV+	SPF cats inoculated with 10^7.2^ TCID_50_ FIV-C36.	3	3	[9]
	SPF-FIV-vx	SPF cats vaccinated with Fel-O-Vax (Boehringer Ingelheim, North Ryde, NSW, Australia) and boosted 2 weeks post prime.	2	0	Unpublished data
	SPF FFV+	SPF cats vaccinated with 2.78 × 10^5^ TCID_50_ FFV pCF-7.	3	3	[8,10]
Field Cats	FIV shelter-adopted cats (FIV-sh)	Cats surrendered to an animal shelter which tested FIV-positive and were rehomed.	9	18	[11]
	Private multi-cat household (MCH)	Cats homed in a large privately owned household.	5	23	[12]

SPF cats were from a closed colony that had been inadvertently exposed to feline parvovirus, and/or had been experimentally infected with FIV or FFV. Field cats consisted of 1 cohort from a shelter setting and a second population from a private multi-cat household. Both populations of field cats were extensively screened for pathogen exposures.

**Table 2:** Field cats on FIV ELISAs produced more indeterminant samples compared to FFV. OD_450_nm average, range, and number of indeterminant samples for each ELISA assay are presented. A significant portion (66.7%) of FIV-negative field cats fell within the indeterminate range compared to FFV field negative samples (7.32%). SPF cats exposed to feline parvovirus and used as Sham cats did not fall within either indeterminate range.

ELISA	Sample Archive	Mean OD_450_	OD_450_ range	Number of Samples in Indeterminant Range
FIV	SPF FPV+ cats	0.095	0.046–0.16	0/6
FIV	SPF Sham cats	0.099	0.067–0.17	0/3
FIV	Field cats	0.23	0.103–0.39	10/15 (66.7%)
FFV	SPF FPV+ cats	0.09	0.04–0.17	0/6
FFV	SPF Sham cats	0.08	0.06–0.12	0/3
FFV	Field cats	0.19	0.04–0.95	3/41(7.32%)

**Table 3:** FIV field cats as compared to SPF and Sham cats were the only groups to produce statistically significant variations in OD values.

	*p*-Value	
	FIV Sample Group	FFV Sample Group
SPF vs Sham cats	0.87	0.66
SPF vs Field negative cats	<0.001	0.098
Sham vs Field negative cats	<0.001	0.425

FIV SPF and Sham cat OD values were not significantly varied. Of note, no statistical differences could be appreciated between SPF or Sham cats compared to FFV field negative cats (using an independent t-test assuming equal variance).

## Data Availability

The data presented in this study will be openly available in Mountain Scholar Digital Collections of Colorado and Wyoming (https://mountainscholar.org/, accessed on 14 May 2021). Currently, uploading of data is not possible due to technical updates taking place on the Mountain Scholar Digital Collections website. Once technical issues have been resolved, data will be published online.
